# Predictors of multiple sexual partnerships and condom use among men engaged in transactional sex: a recursive bivariate probit analysis of Demographic and Health Surveys from 26 Sub-Saharan African countries

**DOI:** 10.1186/s41182-025-00874-7

**Published:** 2025-12-09

**Authors:** Issifou Yaya

**Affiliations:** 1 Unité de Recherche en Santé Des Populations (URESAP), CHU SO Lomé, Lomé, Togo; 2 Patient-Reported Outcomes Research (PROQOL), Health Economics Clinical Trial Unit (URC-ECO), Hotel-Dieu Hospital, AP-HP, Paris, France; 3 ECEVE, UMR-S 1123, Paris Cité University, Inserm, Paris, France

**Keywords:** Transactional sex, Multiple sexual partnerships, Condom use, HIV risk behaviors, Sub-Saharan Africa, Demographic and Health Surveys (DHS)

## Abstract

**Background:**

Men engaged in transactional sex (METS) are a critical population for HIV transmission dynamics in Sub-Saharan Africa (SSA). The interplay between multiple sexual partnerships and condom use, two key risk determinants, is complex and not fully understood. This study aimed to assess the prevalence and determinants of these interdependent behaviors among METS across SSA.

**Methods:**

Cross-sectional data from 26 recent Demographic and Health Surveys (DHS) in SSA were pooled. A sample of 10,128 men who reported providing or receiving money or gifts in exchange for sex was analyzed. A recursive bivariate probit model was use to jointly model the propensity for multiple sexual partnerships and condom use, accounting for their potential correlation. Adjusted coefficients, predicted joint probabilities, and average marginal effects (AME) were reported.

**Results:**

The weighted prevalence of multiple sexual partnerships was 55.3% (95%CI: 53.8–56.9) and of condom use was 32.9% (95%CI: 31.4–34.4). The model revealed a significant, positive correlation between the two behaviors (rho = 0.447, p < 0.05). Key determinants had compensatory effects: living in a couple was associated with a higher propensity for multiple partnerships (β = 0.39, p < 0.001) but a lower propensity for condom use (β = − 0.78, p < 0.001). Higher education was associated with both more multiple partnerships and increased condom use. HIV-related knowledge and testing were strong predictors of condom use but not of multiple partnerships. AME analysis showed that marital status had the largest effect, increasing the probability of the high-risk outcome (multiple partners, no condom) by 25.85 percentage points (p < 0.001). Media exposure and comprehensive HIV knowledge significantly increased the probability of protective behaviors.

**Conclusion:**

Among METS in SSA, there is evidence of risk compensation, wherein factors associated with multiple sexual partnerships are also associated with increased condom use. However, the alarmingly high prevalence of multiple partnerships coupled with low condom use among married METS represents a critical intervention gap.

**Supplementary Information:**

The online version contains supplementary material available at 10.1186/s41182-025-00874-7.

## Background

Sub-Saharan Africa (SSA) remains the region most disproportionately affected by the HIV/AIDS epidemic, accounting for more than two-thirds of people living with HIV worldwide [1]. While significant progress has been made in treatment and prevention, understanding and mitigating the drivers of new infections requires a focused examination of high-risk sexual behaviors. Transactional sex, defined as the non-commercial exchange of money, gifts, or other forms of material support in exchange for sex outside of formal sex work, is a well-documented structural driver of HIV transmission in SSA [2–5].

Much of the existing research on transactional sex has historically focused on women and adolescent girls, typically in contexts of receiving material compensation, often linked to economic vulnerability and gender inequality [6–8]. In contrast, this study focuses on men, who typically occupy the role of providers in these transactions, a perspective that is less commonly examined. Understanding the behaviors of men who engage in transactional sex (METS) is therefore crucial for developing a holistic picture of transmission patterns, informing public health strategies, and addressing the complexities of gender and agency in these interactions. [9, 10]. METS often report higher rates of concurrent sexual partnerships, earlier sexual debut, and a greater number of lifetime partners than men who do not engage in transactional sex [10, 11]. This pattern of multiple sexual partnerships (MSP) exponentially increases sexual network connectivity and the potential for HIV transmission between different population groups [12, 13].

Alongside MSP, condom use is a major determinant of HIV risk. Inconsistent condom use during transactional sex, significantly increases the risk of HIV and other sexually transmitted infections (STIs) [14, 15]. Although one might assume that the explicit “transaction” would lead to more calculated risk management, data suggest that condom use in these settings is inconsistent and influenced by factors such as trust, intimacy, alcohol consumption, and the perceived value of the transaction [16, 17].

Previous research has often examined the determinants of MSP and condom use as separate and independent outcomes [18, 19]. These factors are likely interdependent and influenced by a common set of underlying sociodemographic, economic, and psychosocial factors [20–23]. This approach, however, overlooks a crucial reality: the decision to have multiple partners and the decision not to use a condom may be driven by a shared set of unobserved characteristics. Analyzing these outcomes separately fails to capture this endogeneity and can lead to inefficient estimates and a fragmented understanding of risk profiles [24, 25].

This study aims to address these gaps by conducting a comprehensive analysis of the interdependent determinants of MSP and condom use among METS in sub-Saharan African countries. This study: (1) quantify the prevalence of MSP and condom use among METS; (2) identify their individual and contextual determinants; and (3) assess how these factors jointly influence HIV risk profiles. Our findings provide new insights into the complex interplay between sexual behavior and HIV risk in this population, with important implications for targeted prevention strategies.

## Methods

### Study design and data source

This study is based on a secondary analysis of pooled, repeated cross-sectional data drawn from the Demographic and Health Surveys (DHS) program [26]. The DHS is a long-standing, internationally standardized survey initiative designed to generate reliable, nationally representative data on health, population, and socio-demographic indicators. All DHS surveys are implemented using highly comparable questionnaires and core modules across countries, which ensures consistency and comparability of indicators.

The DHS employs a stratified, two-stage cluster sampling design. In the first stage, primary sampling units (PSUs), usually census enumeration areas (EAs), are selected using probability proportional to size (PPS). In the second stage, a fixed number of households are randomly and systematically drawn within each PSU. All eligible men and women in the selected households are invited to participate, with eligibility criteria based on standard DHS protocols. This complex survey design requires appropriate adjustments in statistical analyses; therefore, we incorporated the sampling weights, stratification, and clustering variables provided in each dataset to produce estimates that are representative at the national and regional levels [27].

For the present analysis, we extracted data from the Men’s Recode (MR) files of DHS surveys conducted in 26 Sub-Saharan African (SSA) countries. Country selection was based on two criteria: (1) availability of a standard DHS survey within the past decade (2011–2021), and (2) inclusion of the sexual behavior module containing the item on transactional sex. A complete list of the included countries, along with survey years and corresponding sample sizes, is presented in Table 1.

**Table 1:** The DHS years of study and study participants of men engaged in transactional sex in twenty-six Sub-Saharan African Countries

Countries	DHS year	Unweighted n (%)	Source of data
Angola	2015–2016	409 (4.0)	https://dhsprogram.com/methodology/survey/survey-display-477.cfm
Benin	2017–2018	408 (4.0)	https://dhsprogram.com/methodology/survey/survey-display-491.cfm
Burundi	2016–2017	121 (1.2)	https://dhsprogram.com/methodology/survey/survey-display-463.cfm
Cameroon	2018	516 (5.1)	https://dhsprogram.com/methodology/survey/survey-display-511.cfm
Chad	2014–2015	141 (1.4)	https://dhsprogram.com/methodology/survey/survey-display-465.cfm
Comoros	2012	121 (1.2)	https://dhsprogram.com/methodology/survey/survey-display-443.cfm
Congo	2011–2012	466 (4.6)	https://dhsprogram.com/methodology/survey/survey-display-388.cfm
DR Congo	2013–14	893 (8.8)	https://dhsprogram.com/methodology/survey/survey-display-421.cfm
Ethiopia	2016	192 (1.9)	https://dhsprogram.com/methodology/survey/survey-display-478.cfm
Gabon	2019–2021	564 (5.6)	https://dhsprogram.com/methodology/survey/survey-display-546.cfm
Gambia	2019–2020	77 (0.8)	https://dhsprogram.com/methodology/survey/survey-display-555.cfm
Guinea	2018	199 (2.0)	https://dhsprogram.com/methodology/survey/survey-display-539.cfm
Liberia	2019–2020	238 (2.4)	https://dhsprogram.com/methodology/survey/survey-display-537.cfm
Madagascar	2021	1,510 (14.9)	https://dhsprogram.com/methodology/survey/survey-display-560.cfm
Malawi	2015–2016	703 (6.9)	https://dhsprogram.com/methodology/survey/survey-display-483.cfm
Mali	2018	164 (1.6)	https://dhsprogram.com/methodology/survey/survey-display-517.cfm
Namibia	2013	46 (0.5)	https://dhsprogram.com/methodology/survey/survey-display-363.cfm
Niger	2012	26 (0.3)	https://dhsprogram.com/data/dataset_admin/index.cfm
Nigeria	2018	660 (6.5)	https://dhsprogram.com/methodology/survey/survey-display-528.cfm
Rwanda	2019–2020	134 (1.3)	https://dhsprogram.com/methodology/survey/survey-display-554.cfm
Sierra Leone	2019	478 (4.7)	https://dhsprogram.com/methodology/survey/survey-display-545.cfm
South Africa	2016	133 (1.3)	https://dhsprogram.com/methodology/survey/survey-display-390.cfm
Togo	2013–14	39 (0.4)	https://dhsprogram.com/methodology/survey/survey-display-328.cfm
Uganda	2016	409 (4.0)	https://dhsprogram.com/methodology/survey/survey-display-504.cfm
Zambia	2018	952 (9.4)	https://dhsprogram.com/methodology/survey/survey-display-542.cfm
Zimbabwe	2015	529 (5.2)	https://dhsprogram.com/methodology/survey/survey-display-475.cfm

### Study population and sample

The source population consisted of all men who were interviewed in the selected DHS surveys. From this source, we constructed our analytical sample by identifying men who reported a lifetime history of transactional sex. Transactional sex was defined according to an affirmative response (“yes”) to the DHS item: “In the last 12 months, have you given gifts or other goods to have sex?” or “Have you been paid for sex in last 12 months”.

This definition captures a wide spectrum of exchanges involving resources for sexual activity. Importantly, it is consistent with the conceptualization of transactional sex as a continuum of relationships, ranging from occasional exchanges to more regular or strategic arrangements, rather than as a binary distinction limited to formal sex work. This broader definition allows for a more nuanced examination of transactional sex as a social and behavioral phenomenon with implications for HIV transmission and other health risks.

**Fig. 1:**
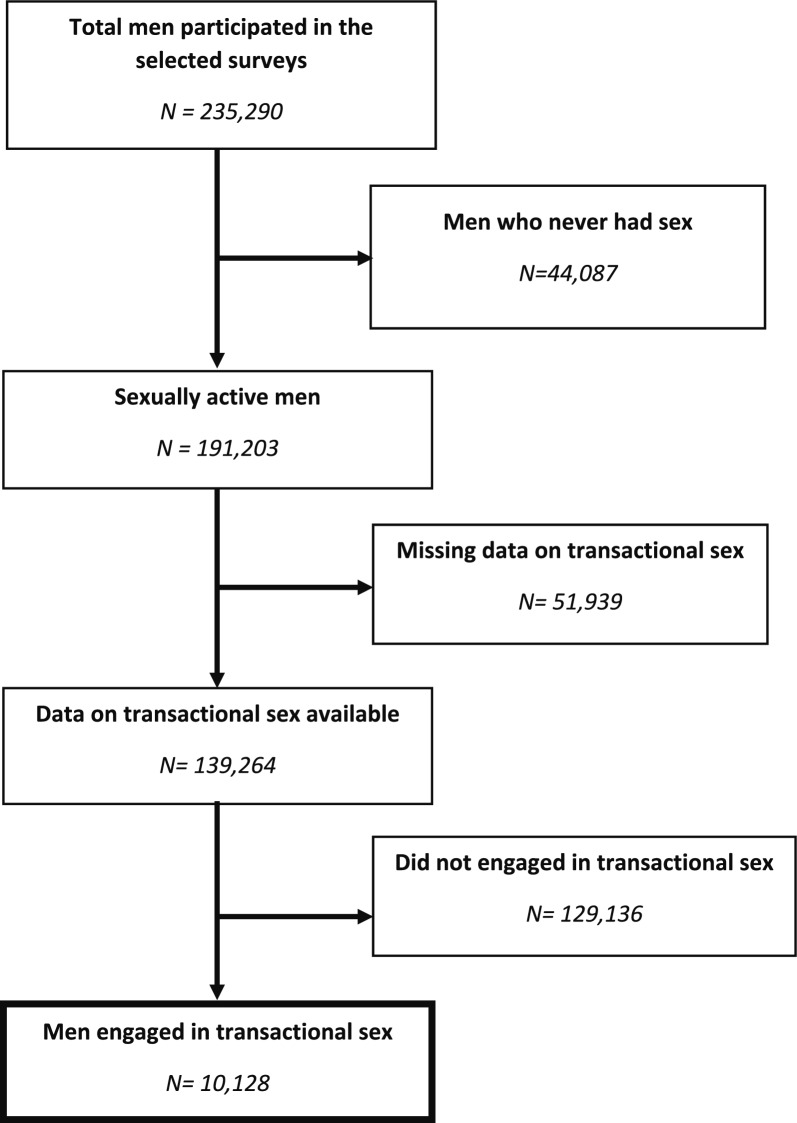
Flow chart of participants’ selection

The selection process for the analytical sample is outlined in the flow diagram (Fig. 1). The initial pooled dataset comprised N = 235,290 men across the 26 countries. Applying the inclusion criterion yielded a final unweighted analytical sample of N = 10,128 men who reported engagement in transactional sex in the last 12 months. All descriptive and inferential analyses applied DHS-provided sampling weights to ensure estimates reflect the population of men engaged in transactional sex across the 26 SSA countries.

### Measurement of variables

#### Outcome variables

We analyzed two distinct but potentially endogenous binary behavioral outcomes, both referencing the 12 months preceding the survey to ensure temporal relevance, reduce recall bias, and align with the timeframe used for defining key populations in UNAIDS guidelines.

- Multiple sexual partnerships (MSP): defined as having had sexual intercourse with more than one woman. This was dichotomized as ‘1’ for two or more partners and ‘0’ for one or zero partners. This measure is a well-established indicator for heightened HIV transmission risk in epidemic modeling [28].
- Condom use at last Sex: this is a widely validated and utilized proxy for *consistent* condom use in large-scale epidemiological surveys like the DHS. While not a perfect measure of consistency, its strong correlation with HIV serostatus makes it a robust and pragmatic outcome. It was dichotomized as ‘1’ if the man reported using a condom during his most recent sexual intercourse and ‘0’ if he did not.

#### Explanatory variables and covariates

Covariates were selected a priori based on a conceptual framework grounded in the socio-ecological model (SEM), which posits that health behaviors are influenced by factors at multiple levels: individual, interpersonal, community, and societal [4].

- **Individual-Level Factors:**
  o *Demographics:* Age group (15–24 years vs. 25 years and older).
  o *Socioeconomic capital:* Educational attainment (categorized as none, primary, or secondary/higher); literacy (defined as the ability to read a whole or part of a sentence: yes/no); current employment status (yes/no).
  o *Health knowledge and behaviors:* Comprehensive knowledge of HIV (yes, if the respondent correctly identified two ways to prevent sexual transmission of HIV and rejected two common local misconceptions); having heard of sexually transmitted infections (STIs) (yes/no); having been tested for HIV in the last 12 months and received the results (yes/no); self-reported circumcision status (yes/no).
- **Interpersonal-Level Factors:**
  o *Partnership status:* Marital or cohabitation status (dichotomized as ‘living in a couple’ or not).
  o *Sexual history:* Age at first sexual intercourse (< 18 years vs. ≥ 18 years).
- **Household-Level Factor:**
  o *Economic status:* The DHS wealth index, a composite measure of a household's cumulative living standard derived via principal component analysis of assets, housing materials, and access to utilities. It was used in its original quintile form but presented in three categories (poorer, middle, richer) for parsimony and interpretability.
- **Community and societal-level factors (Contextual):**
  o *Place of residence:* Urban vs. rural.
  o *Geographic region:* Sub-Saharan African region (Western, Central, Eastern, Southern).
  o *Macro-Level HIV context:* **National HIV prevalence.** We obtained country-specific HIV prevalence estimates for adults (aged 15–49 years) for the year of the survey from the UNAIDS database. These estimates were categorized into three levels to capture the gradient of the epidemic: low (< 1%), medium (1–10%), and high (> 10%).

### Statistical analysis

#### Descriptive analysis

**Fig. 2:**
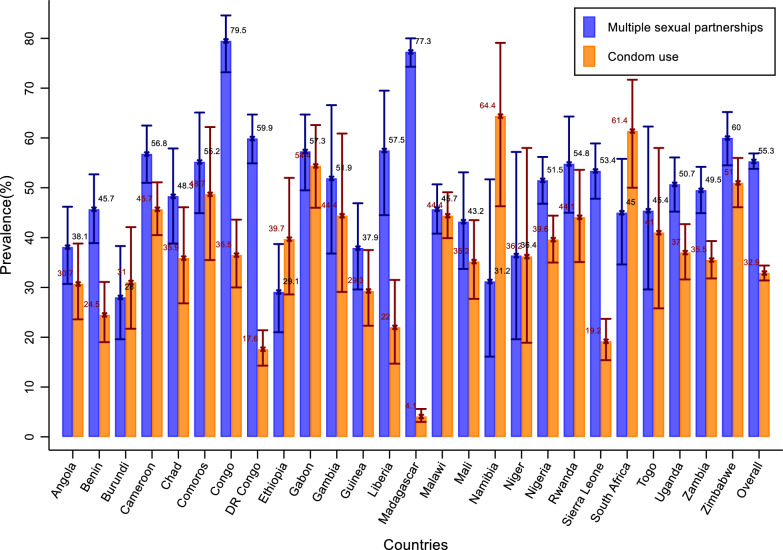
Weighted-prevalence of multiple sexual partnerships and condom use among METS across 26 Sub-saharan African countries

All analyses accounted for the complex survey design using the svy suite of commands in Stata version 15.0 (StataCorp LP, College Station, TX, USA). Sampling weights were applied to ensure representativeness at the pooled regional level. Standard errors were adjusted for the clustering of observations within primary sampling units and for stratification. We computed weighted frequencies and proportions for all categorical variables. The weighted prevalence of MSP and condom use was calculated overall and across all socio-demographic strata. Results are presented in Fig. 2 and Table 2.

**Table 2:** Participants’ characteristics and prevalence of multiple sexual partnerships and condom use among METS in twenty-six Sub-Saharan African Countries

Characteristics (N = 10,128)	Unweighted n (%)	Multiple sexual partnerships		Condom use	
		Weighted prevalence (95%CI)	P-value	Weighted prevalence (95%CI)	P-value
Respondent’s age (years) 15–24 25 and more Mean (± SD)	3811 (37.6) 6317 (62.4) 29.8 (± 10.2)	50.3 [47.8–52.8] 58.3 [56.4–60.2]	** < 0.001**	40.2 [37.8—42.7] 28.7 [26.9–30.5]	** < 0.001**
Education level No education Primary Secondary and higher	1294 (12.8) 3262 (32.2) 5571 (55.0)	48.3 [44.2–52.4] 54.4 [51.9–56.8] 57.5 [55.4–59.6]	** < 0.001**	19.0 [16.0–22.5] 26.8 [24.6–29.0] 39.4 [37.3–41.6]	** < 0.001**
Literate No Yes	2279 (22.5) 7849 (77.5)	50.9 [47.8–54.0] 56.6 [54.8–58.3]	**0.001**	21.9 [19.6–24.5] 36.0 [34.2–37.8]	** < 0.001**
Currently working No Yes	1406 (13.9) 8722 (86.1)	45.5 [41.5–49.7] 56.9 [55.3–58.6]	** < 0.001**	45.6 [41.0–50.4] 30.8 [29.3–32.4]	** < 0.001**
Religious affiliation Traditional/animist/no religion Christianism Islam	6141 (60.6) 2867 (28.3) 1120 (11.1)	55.5 [53.5–57.5] 56.4 [53.5–59.2] 51.2 [46.9–55.4]	0.1410	32.4 [30.5–34.4] 34.3 [31.6–37.1] 32.3 [28.4–36.5]	0.4661
Living in couple No Yes	5606 (55.4) 4522 (44.6)	48.0 [46.0–50.0] 64.5 [62.2–66.7]	** < 0.001**	47.1 [45.0–49.2] 16.0 [14.4–17.9]	** < 0.001**
Households’ wealth index Poorer Middle Richer	3913 (38.6) 2052 (20.3) 4163 (41.1)	53.7 [51.2–56.2] 57.6 [54.4–60.6] 55.4 [53.1–57.8]	0.1730	25.2 [23.1–27.4] 32.4 [28.9–36.0] 38.6 [36.4–40.8]	** < 0.001**
Media exposure No Yes	2086 (20.6) 8031 (79.4)	54.4 [50.8–57.9] 55.5 [53.8–57.2]	0.5490	18.7 [16.2–21.5] 35.8 [34.1–37.6]	** < 0.001**
Age at the first sex Less than 18 years 18 years or more	6309 (62.5) 3781 (37.5)	59.6 [57.6–61.6] 48.0 [45.7–50.3]	** < 0.001**	33.4 [31.5–35.4] 32.3 [30.1–34.6]	0.4422
Comprehensive knowledge about HIV No Yes	6986 (69.0) 3142 (31.0)	55.8 [53.9–57.6] 54.3 [51.7–56.9]	0.3729	29.3 [27.5–31.2] 41.1 [38.5–43.7]	** < 0.001**
Heard about STI No Yes	180 (1.8) 9948 (98.2)	52.8 [40.7–64.5] 55.4 [53.8–56.9]	0.6726	4.8 [2.1–10.5] 33.3 [31.8–34.9]	** < 0.001**
Circumcision No Yes	3384 (33.4) 6744 (66.6)	51.2 [48.5–53.9] 57.4 [55.5–59.3]	** < 0.001**	32.8 [30.5–35.3] 33.0 [31.1–34.9]	0.9345
Tested for HIV** No Yes	7803 (77.0) 2325 (23.0)	55.0 [53.2–56.8] 56.4 [53.5–59.2]	0.4032	29.9 [28.2–31.7] 43.2 [40.2–46.2]	** < 0.001**
Place of residence Urban Rural	3973 (39.2) 6155 (60.8)	55.4 [52.8–57.9] 55.3 [53.4–57.2]	0.9422	41.6 [39.2–44.2] 25.3 [23.6–27.0]	** < 0.001**
Region Western Africa Central Africa Eastern Africa Southern Africa	2289 (22.6) 2701 (26.7) 2366 (23.3) 2772 (27.4)	48.8 [45.4–52.1] 60.9 [57.9–63.9] 64.3 [61.4–67.1] 46.8 [43.8–49.7]	** < 0.001**	28.6 [26.0–31.4] 36.5 [33.5–39.5] 22.4 [19.3–25.9] 43.2 [40.4–45.9]	** < 0.001**
National HIV prevalence < 1 1–10 > 10	1778 (17.6) 6690 (66.0) 1660 (16.4)	68.3 [64.8–71.7] 52.9 [51.0–54.8] 50.7 [47.0–54.4]	** < 0.001**	16.0 [12.3–20.6] 34.6 [32.8–36.3] 47.3 [43.8–50.9]	** < 0.001**

^*^Pearson chi2

^**^in the last 12 months

#### Modeling strategy: recursive bivariate probit model

To efficiently estimate if the error terms of the two outcome equations are correlated due to unobserved confounding, we specified a recursive bivariate probit model, which is a system of two simultaneous equations that estimates the propensity for each outcome while explicitly modeling the correlation between their error terms [29, 30]. The recursive bivariate probit model was estimated using the *svy*: prefix in Stata, which incorporates the sampling weights, accounts for the clustering of observations within primary sampling units, and adjusts for stratification. This ensures that the standard errors of our coefficients are robust to the complex survey design. This model is robust to the endogeneity between the two behaviors, and is formally represented as:

Equation (1): Multiple sexual partnerships

1 $${y}_{1i}^{*}={x}_{1i}{\prime}{\beta }_{1}+{\varepsilon }_{1i}, {y}_{1i}=1 \left[{y}_{1i}^{*}>0\right]$$

Equation (2): Condom use:

2 $${y}_{2i}^{*}={x}_{2i}{\prime}{\beta }_{2}+{\gamma y}_{1i}+{\varepsilon }_{2i}, {y}_{2i}=1 \left[{y}_{2i}^{*}>0\right]$$

With

$$\left(\begin{array}{c}{\varepsilon }_{1}\\ {\varepsilon }_{2}\end{array}\right) \sim N \left(\left(\begin{array}{c}0\\ 0\end{array}\right),\left(\begin{array}{cc}1&\uprho \\\uprho & 1\end{array}\right)\right)$$

where:

- y_1i_^∗^ and y_2i_^∗^ are latent (unobserved) propensities for having MSP and using a condom, respectively.
- y_1i_ and y_2i_ are the observed binary outcomes.
- x_1i_ and x_2i_ are vectors of exogenous covariates.
- β_1_ and β_2_ are vector of coefficients to be estimated.
- γ measures the direct effect of having multiple sexual partners (y_1i_) on the propensity for condom use (y_2i_^∗^).
- ρ (rho) is the correlation coefficient of the error terms. A statistically significant ρ ≠ 0 indicates the presence of unobserved confounding factors (e.g., risk preference, perceived susceptibility) that jointly influence both decisions, validating the use of the bivariate approach [31].

Two sets of models were fitted: (1) crude models: bivariate probit models including only one explanatory variable at a time (unadjusted), (2) final adjusted model: A full multivariate bivariate probit model including all covariates simultaneously to isolate independent associations. Results are presented as coefficients (β) with their robust standard errors. The statistical significance of the correlation coefficient ρ was tested using a Wald test.

Prior to fitting the final multivariate model, we assessed multicollinearity among all covariates by calculating variance inflation factors (VIFs). All individual VIFs were below 10, indicating that multicollinearity was not a substantive concern in our model."

#### Post-estimation interpretation: predicted probabilities and marginal effects

To translate the model coefficients into intuitively meaningful and policy-relevant metrics, extensive post-estimation analysis were conducted:

1. Predicted joint probabilities: We used the predict command post-estimation to calculate the four predicted joint probabilities for each covariate profile, holding all other variables at their mean values:

2. P11: Pr(MSP = 1, Condom use = 1). This represents the probability of "protective" behavior in the context of multiple partnerships.

3. P10: Pr(MSP = 1, Condom use = 0). This represents the high-risk combination.

4. P01: Pr(MSP = 0, Condom use = 1). This represents "preventive" behavior, likely within a primary relationship*.*

5. P00: Pr(MSP = 0, Condom use = 0). This is often the norm in stable, concordant relationships.

These probabilities sum to 1 for each individual and provide a complete picture of their joint behavioral profile.

6. Average Marginal Effects (AME) of each significant covariate on these four joint probabilities were computed using the margins, *dydx()* command. The AME represents the average change in the probability of a joint outcome for a discrete change in a categorical variable, all else held constant. This provides a direct measure of a variable's public health impact.

## Results

### Characteristics of the study’s participants

A total of 235,290 men were included in the selected surveys, among them 139,264 had reported data on transactional sex. Of these, 10,128 men reported having engaged in transactional sex and were included in the analysis (Fig. 1). The weighted prevalence of transactional sex among all men in the 26 countries was 6.5% (95%CI: 6.3–6.8%). The distribution of participants across countries was notably heterogeneous, with the largest contributions to the sample came from Madagascar (n = 1,510; 14.9%), Zambia (n = 952; 9.4%), and the Democratic Republic of Congo (n = 893; 8.8%). Conversely, several countries contributed smaller samples, including Niger (n = 26; 0.3%), Namibia (n = 46; 0.5%), and The Gambia (n = 77; 0.8%). The DHS survey years spanned from 2011 to 2021, with the majority of surveys conducted between 2015 and 2020 (Table 1).

The participants’ characteristics are presented in the Table 2. The mean age was 29.8 years (standard deviation (SD): ± 10.2), with over one-third (37.6%) were aged 15–24 years and nearly two-thirds (62.4%) were 25 years or older. Educational attainment was relatively high, with 55.0% having completed at least secondary school, although 12.8% reported no formal education. Overall, 77.5% of participants were literate, and 86.1% were currently employed. Slightly less than half (44.6%) were living in a couple. Most identified as Christian (60.6%), while 11.1% identified as Muslim and 28.3% as traditional/other. Rural residents represented the majority (60.8%), with regional representation balanced across Western (22.6%), Central (26.7%), Eastern (23.3%), and Southern Africa (27.4%). Nearly two-thirds (62.5%) reported sexual debut before age 18, and one in three (31.0%) demonstrated comprehensive HIV knowledge. While 98.2% had heard of STIs, only 23.0% had been tested for HIV in the past 12 months (Table 2).

Supplementary Table S1 presents a detailed comparison between METS and men not engaged in transactional sex. The data reveal that METS differ markedly from their counterparts: they are significantly more likely to be younger (p < 0.001), employed (p = 0.006), have higher educational attainment (p < 0.001), be circumcised (p < 0.001), live in a couple (p < 0.001), report sexual debut before age 18 (p < 0.001), have comprehensive knowledge about HIV (p < 0.001). This underscores that METS are a distinct subgroup with a specific risk profile.

### Prevalence of multiple sexual partnerships and condom use

The overall weighted prevalence of multiple sexual partnerships was 55.3% (95%CI: 53.8–56.9%). This prevalence was high across the sample but varied by demographic characteristics. Older respondents (≥ 25 years) were more likely than younger participants to report multiple partners, as were those with secondary or higher education and those who were literate. The prevalence was also higher among individuals who were employed, those living in a couple, and those who reported early sexual debut. Regional variation was substantial, ranging from 46.8% in Southern Africa to 64.3% in Eastern Africa. Strikingly, prevalence was highest in countries with national HIV prevalence below 1%, compared to those with higher background prevalence. By contrast, no significant differences were observed by religion, household wealth, media exposure, HIV knowledge, STI awareness, HIV testing, or urban–rural residence (Table 2).

Patterns of condom use differed markedly from those of multiple sexual partnerships. Condom use prevalence was 32.9% (95%CI: 31.4; 34.4%) and was more frequent among younger respondents (15–24 years), participants with higher education, and literate individuals. It was also more common among those not living in a couple, those with greater household wealth, and those with exposure to media. Respondents with comprehensive HIV knowledge, awareness of STIs, or recent HIV testing reported higher levels of condom use than their counterparts. Urban residents were more likely to use condoms than rural residents. Marked regional differences were also observed, with the lowest use recorded in Eastern Africa (22.4%) and the highest in Southern Africa (43.2%). Furthermore, condom use was strongly associated with national HIV prevalence, rising from 16.0% in countries with less than 1% prevalence to 47.3% in those with prevalence above 10% (Table 2).

### Determinants of multiple partnerships and condom use

The model showed a statistically significant correlation coefficient (rho = 0.447, p < 0.05), indicating that the unobserved factors influencing the propensity for multiple partnerships are positively correlated with those influencing the propensity for condom use. This justifies the use of the bivariate approach.

The final adjusted model revealed several significant determinants (Table 3). Socio-demographic characteristics were strongly associated with both outcomes. Men aged 15–24 showed a marginally higher propensity for multiple partnerships (β = 0.05, p < 0.1) and a lower propensity for condom use (β = − 0.03, p < 0.1) compared to older men (25 +). Living in a couple was the strongest predictor, associated with a substantially higher propensity for multiple partnerships (β = 0.39, p < 0.001) and a lower propensity for condom use (β = − 0.78, p < 0.001). Higher educational attainment was also associated with an increased likelihood of both multiple partnerships (Primary: β = 0.15, p < 0.05; Secondary and higher: β = 0.28, p < 0.01) and condom use (Secondary + : β = 0.24, p < 0.05) (Table 3).

**Table 3:** Recursive bivariate probit models for multiple partnerships and condom use in the last 12 months among METS across 26 Sub-saharan African countries

Characteristics (N = 10,128)	Crude effect		Final model		Joint predicted probability (%)				Average marginal effect (%)			
	Multiple sexual partnerships	Condom use	Multiple sexual partnerships	Condom use	P11	P10	P01	P00	P11	P10	P01	P00
Respondent’s age (years) 15–24 25 and more	Ref 0.17***	Ref − 0.32***	Ref 0.05°	Ref − 0.03°	23.7 23.7	33.5 35.1	11.1 10.2	31.8 30.9	Ref 0.03°	Ref 1.67°	Ref − 0.87°	Ref − 0.83°
Education level No education Primary Secondary and higher	Ref 0.12* 0.20**	Ref 0.26*** 0.61***	Ref 0.15* 0.28**	Ref 0.08° 0.24*	18.4 21.3 25.7	32.4 35.1 35.1	10.1 10.3 10.8	38.1 33.2 28.5	Ref 2.96° 7.31***	Ref 2.71° 2.63°	Ref − 0.75° 0.28°	Ref − 4.92* − 9.66***
Literate No Yes	Ref 0.12*	Ref 0.41**	Ref − 0.04°	Ref − 0.004°	24.1 23.6	35.1 34.3	10.3 10.7	30.5 31.4	Ref − 0.49°	Ref − 0.81°	Ref 0.38°	Ref 0.93°
Currently working No Yes	Ref 0.28***	Ref − 0.38***	Ref 0.21**	Ref 0.05°	20.9 24.3	30.6 35.0	12.2 10.2	36.3 30.5	Ref 3.35*	Ref 4.44*	Ref − 1.95	Ref − 5.84**
Religious affiliation Traditional/animist/no religion Christianism Islam	Ref 0.03° − 0.12°	Ref 0.05° − 0.01°	Ref 0.09* − 0.12°	Ref 0.09° − 0.07°	23.2 26.0 20.6	34.5 35.1 32.8	10.6 10.5 11.1	31.8 28.4 35.6	Ref 2.85* − 2.58°	Ref 0.60° − 1.73°	Ref − 0.10° 0.50°	Ref − 3.34* 3.81°
Living in couple No Yes	Ref 0.37***	Ref − 0.91***	Ref 0.39***	Ref − 0.78***	28.9 17.3	22.8 48.7	15.9 3.5	32.4 30.5	Ref − 11.56***	Ref 25.85***	Ref − 12.43***	Ref − 1.86°
Households’ wealth index Poorer Middle Richer	Ref 0.08° 0.06°	Ref 0.21** 0.38***	Ref 0.05° 0.05°	Ref 0.08° 0.10°	22.0 24.1 24.4	34.9 34.6 34.5	10.3 10.7 10.8	32.8 30.6 30.3	Ref 2.10° 2.37°	Ref − 0.30° − 0.40°	Ref 0.39° 0.47°	Ref − 2.19° − 2.41°
Media exposure No Yes	Ref 0.03°	Ref 0.53***	Ref 0.05°	Ref 0.39***	17.4 24.8	39.2 33.7	7.6 11.2	35.9 30.3	Ref 7.42***	Ref − 5.45	Ref 3.62***	Ref − 5.60**
Age at the first sex Less than 18 years 18 years or more	Ref − 0.29***	Ref − 0.03°	Ref − 0.36***	Omitted	25.2 20.8	37.9 28.9	9.1 13.5	27.8 36.8	Ref − 4.36***	Ref − 9.00***	Ref 4.36***	Ref 8.98***
Comprehensive knowledge about HIV No Yes	Ref − 0.04°	Ref 0.32***	Ref 0.02°	Ref 0.24***	22.2 26.9	35.8 31.9	9.8 12.3	32.2 28.9	Ref 4.68***	Ref − 3.89**	Ref 2.49**	Ref − 3.29*
Heard about STI No Yes	Ref 0.03°	Ref 1.24**	Ref 0.06°	Ref 0.82***	10.4 23.8	45.7 34.4	3.8 10.7	40.1 31.1	Ref 13.42***	Ref − 11.30°	Ref 6.86***	Ref − 8.98°
Circumcision No Yes	Ref 0.12**	Ref 0.01°	Ref 0.03°	Ref 0.17***	21.4 24.8	36.1 33.7	9.6 11.3	32.9 30.2	Ref 3.46**	Ref − 2.37°	Ref 1.60*	Ref − 2.69°
Tested for HIV^a^ No Yes	Ref 0.01°	Ref 0.35***	Ref 0.03°	Ref 0.21***	22.6 26.9	35.3 32.2	10.2 12.2	31.9 28.7	Ref 4.27**	Ref − 3.15*	Ref 2.05**	Ref − 3.17*
Place of residence Urban Rural	Ref 0.002°	Ref − 0.46***	Ref − 0.03°	Ref − 0.18**	25.5 21.8	33.3 35.9	11.5 9.8	29.8 32.5	Ref − 3.63*	Ref 2.65°	Ref − 1.73*	Ref 2.72°
Region Western Africa Central Africa Eastern Africa Southern Africa	Ref 0.25*** 0.35*** − 0.09°	Ref 0.22*** − 0.19** 0.39***	Ref 0.14* 0.09° − 0.36***	Ref 0.25*** 0.18* 0.21*	21.2 27.7 25.7 20.4	38.2 36.8 37.0 25.3	8.2 9.2 9.0 15.2	32.4 26.4 28.2 39.2	Ref 6.43*** 4.50* − 0.81°	Ref − 1.36° − 1.13° − 12.9***	Ref 1.01° 0.87° 7.01***	Ref − 6.08** − 4.24° 6.72*
National HIV prevalence (%) < 1 1–10 > 10	Ref − 0.37*** − 0.44***	Ref 0.60*** 0.93***	Ref − 0.27** 0.003°	Ref 0.32** 0.48***	20.0 23.4 29.9	45.0 31.7 35.0	5.7 11.6 10.1	29.3 33.3 25.0	Ref 3.36° 9.90**	Ref − 13.23*** − 10.01**	Ref 5.89*** 4.37**	Ref 3.98° − 4.26°
rho			0.447*									

^a^in the last 12 months;

Significant at: ° p < 0.1; *p < 0.05; **p < 0.01; ***p < 0.001

P11 = Probability that a participant who had multiple partners in the last 12 months has also used condom consistently. P10 = Probability that he had multiple partners but did not use condom consistently. P01 = Probability that he did not have multiple partners and has used condom consistently. P00 = Probability that he did not have multiple partners but did not use condom consistently

HIV-related knowledge and behaviors were significant drivers of condom use but not of multiple partnerships. Comprehensive HIV knowledge (β = 0.24, p < 0.001), having heard about STIs (β = 0.82, p < 0.001), and having been tested for HIV in the past 12 months (β = 0.21, p < 0.001) were all associated with a significantly higher propensity for consistent condom use (Table 3).

Contextual factors also played a significant role. Men residing in urban areas had a lower propensity for condom use than those in rural areas (β = − 0.18, p < 0.01). Significant regional variations were observed: compared to Western Africa, men in Eastern Africa had a higher propensity for multiple partnerships (β = 0.14, p < 0.05), while men in Southern Africa had a significantly lower propensity (β = − 0.36, p < 0.001). National HIV prevalence was a key contextual determinant. Residing in a high-prevalence (> 10%) country was strongly associated with a higher propensity for condom use (β = 0.48, p < 0.001) compared to living in a low-prevalence (< 1%) country (Table 3).

### Predicted joint probabilities and average marginal effects

The joint predicted probabilities (P11, P10, P01, P00) estimate the likelihood of each combination of behaviors for a given characteristic, holding all other variables constant. The *P11 (MSP and condom use)* was highest (29.9%) for men living in country with higher HIV prevalence (> 10%) and lowest for those who did not hear about STI (10.4%). The *P10 (MSP and no condom use)* was alarmingly high for men living in a couple (48.7%), indicating that nearly half of cohabiting METS with multiple sexual partners did not use condoms consistently, posing a extreme risk for HIV transmission within and beyond their primary relationship. The *P01 (One partner and condom use)*: METS who were not living in couple had a notably higher probability of this outcome (15.9%) compared to those who were (3.5%). The *P00 (One partner and no condom use)* was the most likely outcome for METS who did not hear about STIs (40.1%), those residing in Southern Africa (39.2%). and those with no education (38.1%) (Table 3).

### Average Marginal Effects (AME)

The analysis of Average Marginal Effects (AME) provides a nuanced understanding of how each covariate influences the joint probabilities of multiple partnership patterns and condom use, holding all other variables constant. The results were presented in Table 3.

Higher education (secondary and higher) was associated with a 7.31 pp increase (p < 0.001) in the P11 probability and a − 9.66 pp decrease (p < 0.001) in the P00 (one partner, no condom) probability. Employment status significantly altered risk profiles. Being employed was associated with a 3.35 pp increase (p < 0.05) in the protective P11 outcome but also a 4.44 pp increase (p < 0.05) in the high-risk P10 outcome, alongside a 5.84 pp decrease (p < 0.01) in the low-risk P00 outcome. Religious affiliation also played a role; adherence to Traditional/animist/no religion was associated with a 2.85 pp increase (p < 0.05) in the P11 outcome and a 3.34 pp decrease (p < 0.05) in the P00 outcome compared to Islam. Living in a couple had the most profound effects, drastically increasing the high-risk P10 outcome (+ 25.85 pp, p < 0.001) and decreasing protective behaviors (P11: − 11.56 pp, p < 0.001; P01: − 12.43 pp, p < 0.001). An early sexual debut (before 18) was associated with a major 9.00 pp decrease (p < 0.001) in the high-risk P10 outcome but a 4.36 pp increase (p < 0.001) in the preventive P01 outcome. Media exposure was associated with a shift towards safer practices, increasing the probability of the P11 (multiple partners, used condom) outcome by 7.42 pp (p < 0.001) and the P01 outcome by 3.62 pp (p < 0.001) (Table 3).

Circumcision was associated with a modest but significant shift towards protection, increasing the P11 (+ 3.46 pp, p < 0.01) and P01 (+ 1.60 pp, p < 0.05) probabilities. Comprehensive HIV knowledge was associated with a significant 4.68 pp increase (p < 0.001) in the protective P11 outcome and a 2.49 pp increase (p < 0.01) in the preventive P01 outcome. This was mirrored by a decrease in the high-risk P10 (− 3.89 pp, p < 0.01) and low-risk P00 (− 3.29 pp, p < 0.05) probabilities. Similarly, having heard of STIs had the largest positive effect on condom use, increasing the probabilities of P11 (+ 13.42 pp, p < 0.001) and P01 (+ 6.86 pp, p < 0.001). Having tested for HIV in the last 12 months was associated with a similar, though less pronounced, shift towards protected sex, increasing P11 (+ 4.27 pp, p < 0.01) and P01 (+ 2.05 pp, p < 0.01) while decreasing unprotected outcomes (Table 3).

Regionally, residing in Central Africa (ref: Western Africa) was associated with a 6.43 pp increase (p < 0.001) in the P11 outcome and a 6.08 pp decrease (p < 0.01) in the P00 outcome. Conversely, Southern Africa was associated with a massive 12.9 pp decrease (p < 0.001) in the high-risk P10 outcome but a 7.01 pp increase (p < 0.001) in the preventive P01 outcome. National HIV prevalence was a key structural driver; living in a country with high prevalence (> 10%) was associated with a 9.90 pp increase (p < 0.01) in the protective P11 outcome and a corresponding 10.01 pp decrease (p < 0.01) in the high-risk P10 outcome compared to countries with low prevalence (< 1%) (Table 3).

## Discussion

This study provides one of the most comprehensive multicountry analyses of sexual risk behaviors among men who reported transactional sex (METS) in the last 12 months across sub-Saharan Africa. Using harmonized DHS data, we investigated the prevalence and determinants of multiple sexual partnerships and condom use, as well as their joint distribution. The demographic profile revealed a predominantly young (mean age 29.8 years), literate, and employed cohort, with a significant proportion living in rural areas and identifying as Christian. Despite high STI (including HIV) awareness (98.2%), only 23.0% had undergone recent HIV testing, highlighting a gap between knowledge and preventive action. It is important to note that METS in our sample are not representative of the general male population in SSA but represent a distinct subgroup with specific sociodemographic profiles (see Supplementary Table S1). This should be considered when generalizing these findings.

Three major findings emerge. First, multiple partnerships were highly prevalent among METS, while consistent condom use remained comparatively low. Second, socio-demographic and behavioral factors shaped these outcomes in distinct ways, with living in a couple and educational attainment exerting particularly strong influences. Third, contextual drivers; including regional location and national HIV prevalence, were strongly associated with both protective and high-risk behavioral patterns.

High prevalence of multiple sexual partnerships (55.3%) confirms that METS are a high-risk population. This aligns with research showing that men's control over economic resources and social freedom contributes to concurrency across the region [32, 33]. Concurrency is well-documented as a driver of HIV transmission in sub-Saharan Africa [34, 35]. Consistent with previous findings, higher education, literacy, employment, and living in a couple are associated with increased multiple sexual partnerships [13, 20]. This suggests that socioeconomic empowerment can facilitate concurrency, possibly due to increased mobility or reduced social constraints.

Condom use (32.9%) was substantially lower than the level required to mitigate risk, and its determinants diverged from those of multiple partnerships. Youth, educated and literate men, those with HIV awareness or recent testing, and wealthier or urban individuals reported higher use. This mirrors patterns seen in other studies among youth in Malawi [36] and meta-analyses of consistent condom use, education [37], HIV awareness, testing, and media exposure are consistent facilitators. The strong positive association between comprehensive HIV knowledge, STI awareness, recent testing, and condom use supports the effectiveness of information-based interventions [38]. In a systematic review of MSM in sub-Saharan Africa, knowledge and self-efficacy correlated with condom use [37].

The most striking finding of this study is the paradoxical nature of primary partnerships. Men living in a couple were significantly more likely to have multiple sexual partners but dramatically less likely to use condoms. Our model predicts that nearly half (48.7%) of cohabiting METS with multiple partners do not use condoms, a behavioral pattern that poses a substantial risk for HIV transmission to their primary partners and within broader sexual networks. This finding aligns with a growing body of evidence suggesting that stable relationships can, counter-intuitively, become epicenters of HIV risk due to a phenomenon known as "concurrency" [39–41]. Within a primary partnership, perceptions of trust and intimacy may lead to the abandonment of condoms, a practice that is not re-negotiated even when outside partners are introduced [42, 43]. This "condom-less contract" within the main relationship creates a highly efficient bridge for STI transmission from external sexual encounters [44]. Our results powerfully suggest that HIV prevention programs must shift focus to include men in seemingly stable relationships, addressing the specific challenges of negotiating condom use within the context of concurrent partnerships.

Consistent with established health behavior theories, our findings reaffirm the protective shield afforded by knowledge and socioeconomic advancement. Higher educational attainment, comprehensive HIV knowledge, and media exposure were all strong predictors of consistent condom use. This underscores the enduring success of public health strategies centered on education and information dissemination. The independent association of higher education with increased condom use, even after adjusting for comprehensive HIV knowledge, suggests that education's influence extends beyond the mere acquisition of health information. It likely fosters general cognitive skills, economic empowerment, and self-efficacy that enable individuals to act on their knowledge and negotiate safer sex practices [45]. This underscores that while information campaigns are vital, broader investments in education can have powerful, synergistic effects on health behavior. Similarly, engagement with health services, such as recent HIV testing, was a powerful correlate of safer sex [46]. This suggests that touchpoints with the healthcare system provide critical opportunities for risk-reduction counseling and reinforce the importance of integrating HIV prevention into routine medical care [47].

Finally, our analysis demonstrates that individual sexual decision-making is profoundly shaped by the macro-level context. The finding that condom use is significantly higher in countries with a high national HIV prevalence points to a rational behavioral response to a heightened risk environment. This aligns with the concept of the "health belief model," where perceived susceptibility to a threat is a key driver of behavioral change [48]. Furthermore, the substantial regional variations, particularly the lower-risk profile observed in Southern Africa despite its mature epidemic, highlight the impact of long-term, intensive prevention campaigns and differing socio-cultural norms on sexual behavior [1]. These contextual effects underscore the need to tailor strategies to the specific epidemiological and cultural realities of a given region or country [49, 50].

The use of a recursive bivariate probit model was a key strength of this study, allowing us to account for the endogeneity between the two risk behaviors. The significant positive correlation of the error terms suggests that unobserved traits, such as an underlying propensity for risk-taking, may jointly influence both the likelihood of having multiple partners and the decision to use condoms. By modeling these outcomes simultaneously, we were able to provide a more holistic and efficient picture of the determinants of sexual risk profiles.

Several limitations should be considered when interpreting these findings. The cross-sectional nature of the DHS data precludes causal inference. Furthermore, our reliance on self-reported sexual behavior may be subject to social desirability bias, potentially leading to an underestimation of risk behaviors. The broad definition of "transactional sex" may also mask significant heterogeneity within this key population. Furthermore, our measure of “condom use at last sex” does not specify the type of partnership in which it occurred. The lower condom use among married men could reflect norms within the primary relationship, even if they used condoms with casual or transactional partners. This ambiguity should be considered when interpreting the results. Finally, while we controlled for regional and national factors, considerable cultural and policy variations exist within these large geographic areas.

## Conclusion

This multicountry analysis underscores that METS in sub-Saharan Africa represent a heterogeneous but consistently high-risk population. While multiple partnerships are common, condom use remains inadequate, particularly among men in cohabiting unions. Educational attainment, HIV knowledge, and service engagement foster condom use, but structural and contextual factors profoundly shape risk profiles. By adopting a joint behavioral modeling approach, this study provides new insights into the interplay of concurrency and condom use and highlights the urgent need for context-sensitive, couple-focused, and structurally informed HIV prevention strategies.

## Supplementary Information

Additional file 1.

## Data Availability

Data is available online from the “measures DHS program” and taken after writing a concept note and getting permission to use it. Anyone can access this data set by registering through this website [https://dhsprogram.com/data/new-user-registration.cfm].
