# Supplementary material for: Predictors of multiple sexual partnerships and condom use among men engaged in transactional sex: a recursive bivariate probit analysis of Demographic and Health Surveys from 26 Sub-Saharan African countries
Source: Trop Med Health. 2025 Dec 9;53:183. doi: 10.1186/s41182-025-00874-7 (PMC12690923; doi:10.1186/s41182-025-00874-7)
Supplement: Supplementary file 1 — Additional file 1. [file 41182_2025_874_MOESM1_ESM.docx]

**Supplementary Table S1** : Comparison the characteristics of men who engaged in transactional sex (our analytical sample, N=10,128 ) with those who did not (N= 129,136)

| **Characteristics (N= 139,264)** | **METS** | | **p-value*** |
| --- | --- | --- | --- |
|  | **Yes (N=10,128)**  **n (%)** | **No (N= 129,136)**  **n (%)** |  |
| **Respondent’s age** (years)  15–24  25 and more  Mean (±SD) | 3811 (37.6)  6317 (62.4)  29.8 (±10.2) | 28751 (22.3)  100385 (77.7)  34.7 (±11.5) | **<0.001** |
| **Education level**  No education  Primary  Secondary and higher | 1294 (12.8)  3262 (32.2)  5571 (55.0) | 26888 (20.8)  38022 (29.5)  64213 (49.7) | **<0.001** |
| **Literate**  No  Yes | 2279 (22.5)  7849 (77.5) | 34942 (27.1)  94194 (72.9) | **<0.001** |
| **Currently working**  No  Yes | 1406 (13.9)  8722 (86.1) | 19231 (14.9)  109901 (85.1) | **0.006** |
| **Living in couple**  No  Yes | 5606 (55.4)  4522 (44.6) | 39117 (30.3)  90019 (69.7) | **<0.001** |
| **Religious affiliation**  Traditional/animist/no religion  Christianism  Islam | 6141 (60.6)  2867 (28.3)  1120 (11.1) | 76619 (59.3)  33843 (26.2)  18674 (14.5) | **<0.001** |
| **Households’ wealth index**  Poorer  Middle  Richer | 3913 (38.6)  2052 (20.3)  4163 (41.1) | 51174 (39.6)  24911 (19.3)  53051 (41.1) | **0.032** |
| **Media exposure**  No  Yes | 2086 (20.6)  8031 (79.4) | 26615 (20.6)  102301 (79.4) | 0.950 |
| **Age at the first sex**  Less than 18 years  18 years or more | 6309 (62.5)  3781 (37.5) | 52806 (42.2)  72441 (57.8) | **<0.001** |
| **Comprehensive knowledge about HIV**  No  Yes | 6986 (69.0)  3142 (31.0) | 81843 (63.4)  47293 (36.6) | **<0.001** |
| **Heard about STI**  No  Yes | 180 (1.8)  9948 (98.2) | 2923 (2.3)  126195 (97.7) | **0.001** |
| **Circumcision**  No  Yes | 3384 (33.4)  6744 (66.6) | 49140 (38.1)  79996 (61.9) | **<0.001** |
| **Risky sexual behavior**  No  Yes | 1887 (18.6)  8241 (81.4) | 28463 (22.0)  100673 (78.0) | **<0.001** |
| **Tested for HIV****  No  Yes | 7803 (77.0)  2325 (23.0) | 99691 (77.2)  29445 (22.8) | 0.721 |
| **Place of residence**  Urban  Rural | 3973 (39.2)  6155 (60.8) | 49254 (38.1)  79882 (61.9) | 0.587 |
| **Community poverty levels**  Low  High | 5099 (50.4)  5029 (49.6) | 60945 (47.2)  68191 (52.8) | **<0.001** |
| **Community literacy level**  Low  High | 7199 (71.1)  2929 (28.9) | 80417 (62.3)  48719 (37.7) | **<0.001** |
| **Level of media exposure**  Low  High | 3813 (37.6)  6315 (62.4) | 58516 (45.3)  70620 (54.7) | **<0.001** |
| **Region**  Western Africa  Central Africa  Eastern Africa  Southern Africa | 2289 (22.6)  2701 (26.7)  2366 (23.3)  2772 (27.4) | 40304 (31.2)  30586 (23.7)  25536 (19.8)  32710 (25.3) | **<0.001** |
